# Sustainable effects of a motor skill programme on physical activity levels in 7–8 years old children, in the Eastern Cape Province of South Africa

**DOI:** 10.1186/s12887-024-04845-5

**Published:** 2024-05-29

**Authors:** Mere Idamokoro, Anita Elizabeth Pienaar, Barry Gerber, van Gent Maya

**Affiliations:** 1 Focus Area of Physical Activity, Sport and Recreation (PhASRec), Faculty of Health Sciences, North West University, Potchefstroom, Republic of South Africa; 2 Department of Human Movement Science, University of Fort Hare, Alice, Republic of South Africa

**Keywords:** Sustainable, Motor skills program, Physical activity, School children

## Abstract

**Background:**

Deteriorating global physical activity (PA) levels among children warrants new and sustainable approaches to increase PA levels. This study aimed to determine the immediate and sustainable influences of a 9-week movement program on the PA levels in 7 to 8-year-old school children in the Raymond Mhlaba Municipality in the Eastern Cape Province of South Africa.

**Methods:**

A randomized control ﻿trial including two groups (control group (CG) and intervention group (IG)), pre–post–retest (after six months of no intervention) design was used. Seventy school children, mean age 7.12 years (± 0.71) (*n* = 35 IG; *n* = 35 CG) participated in the study. A 9-week movement program was followed twice a week for 30 min during school hours. PA was measured for 7 consecutive days using a hip-mounted wGT3X-BT Actigraph accelerometer. The Test of Gross Motor Development-Third Edition (TGMD-3) was used to assess motor skills. Hierarchical Linear Modelling (HLM) was applied to analyze the data with time, sex, and group as predictors. Effect sizes were computed using Cohen’s d-cut points to assess the practical significance of changes over time. Estimated regression coefficients were also computed to determine the strength of the relationship between moderate-to-vigorous physical activity (MVPA) and fundamental movement skills (FMS).

**Results:**

Before the intervention, 60% of the IG met the 60 min of daily MVPA guideline, while light physical activity (LPA) per day was also higher than sedentary behavior (SB) in both groups. No immediate (*p* < 0.01) or sustainable (*p* < 0.01) increases in MVPA levels were found and no positive associations emerged between FMS and MVPA levels.

**Conclusions:**

This intervention had little to no effect on children’s MVPA. More understanding of the activity behavior and interests of children is needed to improve their PA behavior through the content of movement programs. Strategies are also needed to communicate clear messages at a personalized but also parental level, focusing on enhancing health through regular PA, especially to promote PA in young children.

## Background

Lower intensities of physical activity (PA) and high levels of sedentary behavior (SB) are reported globally among 5- to 17-year-old children and adolescents [1]. The Global Matrix 4.0 Physical Activity Report Card Grades of 2022 involves 57 countries, reported a global average grading for PA as a D. This grade implies that only 20–40% of children reached the recommended World Health Organization (WHO) guidelines of 60 min of moderate-to-vigorous-physical-activity (MVPA) daily [1]. A current narrative review by Aubert et al. [2]. also confirmed insufficient levels of PA among children worldwide, with lower PA levels in girls compared to boys of the same age. PA also continues to decline with increasing age. In recent times, the impact of the COVID-19 lockdown has also negatively impacted children’s PA as the ability for children to engage in outdoor play was notably diminished, and the complete closure of schools eliminated physical education (PE) classes as schools were completely closed [3].

The World Health Organization [4] suggests that, regardless of sex, race, ethnicity, or socio-economic status, children and adolescents aged 5–17 years should accrue at least 60 min of MVPA daily for health benefits while also acknowledging that exceeding 60 min daily would provide additional health benefits. Vigorous-intensity aerobic activities and exercises that strengthen the muscles and bones should also be incorporated at least 3 days per week to improve cardiorespiratory and muscular fitness, bone health, and metabolic health biomarkers, and reduce symptoms of anxiety and depression [4–6].

In South Africa (SA), a recognizable shift towards physical inactivity and obesity in the general population has been experienced and is attributed to the process of urbanization and associated socio-economic change [7]. The Global Matrix 4.0 Physical Activity Report Card of 2022, however, reported a B- grade for overall PA among SA children and adolescents portraying that 60–66% were meeting the minimal daily requirements of MVPA [1]. This is promising compared to previous reports but should be considered in the context that very few studies were recently published on the PA levels of SA children. On the other hand, a lower C- grade is reported for sedentary behavior (SB), and since the onset of the COVID-19 pandemic in early 2020, rapid shifts in lifestyle and decreases in PA levels have been observed [8]. Movement and recreational access were limited due to restrictions, leading to an environment conducive to weight gain. This, in turn, escalated health risk factors like obesity among children in SA [8].

In current years, the school setting has received special consideration in the field of health promotion, and interventions have been carried out in this regard [9]. Schools represent an available platform in which engagement in PA can be stimulated because of the existing vital link with the school curriculum and the possibility of directly reaching all children and youth through Physical Education (PE) [10]. Similarly, activity interventions within the school setting can promote positive learning experiences, complement PE, and therefore increase motivation-based exercise, which may improve and influence children’s and adolescent’s total PA levels and health [11]. This argument was supported by Messing et al. [12]. who identified several promising strategies for promoting PA among children and adolescents. In their review, the school environment was recognized as the most promising strategy to support PA participation among school children through intervention programs. Lonsdale et al. [13]. explained that interventions delivered in the school setting during PE lessons can increase children’s MVPA by about 24%, therefore influencing their total daily amount of PA. Additionally, 129 studies were reviewed by Demetriou and Höner [14] regarding the effectiveness of interventions in school settings among children aged 12 years and younger. This review reported that the duration of interventions ranged from less than three months to 13 or more months and the frequency from once to four times per week. Approximately 56.8% of these studies that measured PA achieved a significant positive effect in favor of the intervention group, also confirming that the school setting serves as a reliable approach to promoting children’s health through school-based health promotion programmes [15].

Furthermore, a narrative review by Lopes et al. [16], revealed that one of the foundations of a physically active lifestyle, which should be focused on during school-based health promotion programs, is motor competence (MC). In this regard, especially during the middle and later childhood years, higher levels of MC are found to offer a greater motor repertoire to engage in various physical activities, games, and sports, resulting in higher PA behavior among children and adolescents [17]. Consequently, children who are moderately to highly skilled will likely self-select higher levels of PA, while low proficient levels of MC children will be involved in lower levels of PA [17]. This highlights the possibility that MC might drive PA levels [17]. A recent systematic review by Barnett et al. [18], however, provides evidence that still shows inconsistent evidence for an MC to PA pathway as patterns of relationships between MC and PA ranged from strong evidence for total MC to no evidence for object control skills. Further research was recommended by these researchers to determine which of the MC skill domains can promote PA.

The understanding of the possible link between fundamental movement skills (FMS), which is considered to be the building blocks of MC, and PA across the lifespan, might therefore be key in contributing to a possible rise in overall PA participation among children and adolescents [16]. Lopes et al. [16], reported that although basic FMS patterns can be developed through free play, mature forms of MC, which refer to the most proficient mastery level of executing FMS, must be encouraged by PA participation but also achieved through appropriate practice, encouragement, feedback, and instruction which is only achievable through appropriate intervention programmes. Competence in FMS, therefore, seems to be considered a criterion for involvement in PA and sports participation, and should therefore receive the necessary attention during the early grades of primary schools through their Physical Education (PE) programs.

Unfortunately, within the current school education systems, the time allocated for PE or even the lack of any time allocated for PE, where competence in and mastery of all FMS should be addressed, is a challenge [11], which results in inadequate time for improving FMS. According to a national research study conducted by the South African University Physical Education Association (SAUPEA), 9.1% of public primary schools in the nine Provinces of SA recorded no regular implementation of PE. More so, PE is also less frequently available in disadvantaged primary schools where reduced sports participation and impaired motor competence are reported as a result [19]. In the Eastern Cape province, one of the poorest provinces in SA, where this study was conducted, the highest percentage of schools are classified as disadvantaged schools where 27.3%, 24.7%, and 19.6% of schools are respectively classified as quintiles 1, 2, 3 schools, and 17%, and 11.4% as quintile 4, and 5 schools. Quintile 1 to 3 schools are the lowest-ranking schools where children are exempt from paying school fees, food schemes are implemented, and infrastructure is poor with a general lack of facilities and qualified teachers to foster PA and sports participation [20]. The highest percentage (16.8%) of primary schools with irregular implementation of PE is reported to be quintile 1 schools [19].

Despite the efficacy of PA intervention programs that are confirmed by review studies, there is still limited research on the sustainability effects of such interventions [21]. In addition, most existing PA intervention studies on children have been conducted in developed countries, which may not be suitable to generalize its implementation to less developed countries or disadvantaged areas. Very few intervention studies have been conducted in SA over the past years to determine the PA levels of school children and adolescents [22–24], especially in the age ranges of 9–12 years. The sustainability effects were also not reported and the possible association between MC and PA was also not investigated. A few PA intervention studies have been conducted by researchers in early childhood and preschool settings [25, 26] but none of these studies focused on the enhancement of PA through the improvement of motor skills in school children growing up in disadvantaged areas of SA. The degree of the relationship between PA and gross motor competence is also still not clear and needs more understanding. The age period of 7 to 8 years is furthermore considered a key period to develop mature patterns of FMS as a foundation for a physically active lifestyle and to profit children with a variety of physiological, social, and cognitive health benefits later in their lives [18, 27]. Limited to no motor intervention studies have reported sustainability effects, especially among primary school children in disadvantaged communities [22]. This lack of understanding from existing studies on the associations between MC and PA, and especially the possible sustainable effects of improving MC on the PA of developing children in disadvantaged settings, motivates the aim of the current study. This study therefore aims to determine the immediate and sustainable effects of a motor intervention on the PA levels of 7 to 8-year-old school children growing up in a rural, disadvantaged area of the Eastern Cape of South Africa.

## Methods

### Research design

The randomized control trial study was based on a two-groups (control and intervention group) pre–test, post–test and re-test design to evaluate the influence of a 9-week movement program on the PA levels of 7 to 8-year-old schoolchildren, growing up in a disadvantaged area of the Raymond Mhlaba Municipality in the Eastern Cape of South Africa. The 9-week fundamental movement skills program was presented by the researcher during school hours twice per week (thus 18 lessons) for 30 min to the intervention group. Practical reasons had to be taken into consideration when planning the frequency and duration of the intervention program. South Africa has 4 school terms per school year and one school term last 12 weeks. Three of these 12 weeks were needed for recruiting, pre-and post-testing, and wearing of Actigraphs, otherwise, the school holidays would have interfered with the results. The study commenced in January 2022 when the schools in South Africa started the first school term. The participants in the intervention group were fitted with Actigraphs immediately after completing the pretesting of their motor skills on one school day and then started the program immediately after 7 days of wearing the Actigraphs. The immediate or post-test effect of the program on the PA levels of both groups was determined immediately after completing the program at the end of March by fitting them with Actigraphs for seven days just before the school holidays started. Both groups were again retested and fitted with Actigraphs at the end of September after six months of no intervention just before schools closed for the October recess to determine the possible sustainable effects of the program on the PA levels of the participants.

### Study population

Children in Grade Two (7- to 8-year-old) attending primary schools in Alice were the target population for this study. Within Alice Town, three of seven public primary schools were randomly selected and invited to be involved in the study and consent for participation. All three schools had quantile 3 rankings, were located far apart in disadvantaged settings in and around Alice, and were considered to be equal in demographics and facilities. The number of participants to be involved in this study was determined by a power analysis through Statistica for Windows [28]. A parent meeting was held before the start of the study at the different schools to inform the parents of the study objectives, and to invite them to enroll their children in the study, and they were also educated on the correct wearing of the Actigraphs. The total number of participants that were recruited, taking age and sex into account was one hundred and six, but only ninety-three consented, representing a dropout rate of 12.2% at the recruitment stage. All the children and their parents voluntarily participated in this study with the parent’s signed informed consent. One intervention school was selected based on the higher number of participants who were willing to participate in the study and two schools were needed for the control group to match the number of participants that had to be recruited for the intervention. Parental consent and verbal child assent were obtained from sixty children who were recruited for the intervention group. However, only thirty-five of the parents of the intervention group consented for their children to wear an Actigraph. This decision is most probably based on cultural influences. These include 17 boys and 18 girls at the pre-test and post-test. At the re-test, one participant had left the school leaving 34 with complete actigraph data (*n* = 17 boys; *n* = 17 girls).

Forty-six children could be recruited for the control group from the two remaining schools. Ten of them did not receive parental consent or were above the age limit leaving thirty-six children (*n* = 17 boys; *n* = 19 girls) with a mean age of 7.36 (*±* 0.68) years that were eligible for participation in the study. Thirty-five participants (*n* = 17 boys; *n* = 18 girls) wore the Actigraph at the pre-test as one was absent when fitted with the Actigraphs. The number of participants that were fitted with Actigraphs reduced to 32 (*n* = 17 boys; *n* = 15 girls) at post-testing and to thirty (*n* = 14 boys; *n* = 16 girls) at retesting.

### Description of instruments and measurements

#### Physical activity as measured with actiGraph accelerometers

The PA of each participant was measured for seven consecutive days using the hip-mounted wGT3X-BT Actigraph accelerometer (Fort Walton Beach, FL, USA). Accelerometers have been validated for evaluating children’s PA in the laboratory and field setting [29, 30]. Each participant received an instruction sheet for parents or guardians, which contained directives on the proper usage of the monitor. Instructions regarding the proper usage of the accelerometer were also explained to all the participants. The monitor had to be put on during waking hours and should only be removed during bathing, showering, or swimming. The age and sex of the children were recorded, and an identifying code was paired with an accelerometer that was assigned to each child. The hip-mounted Actigraph accelerometer was fitted at the waist of a child just over the right mid-axillary line, according to the directives of the manufacturer. A trained research assistant showed the correct positioning of the monitor and how to clip and unclip the monitor elastic belt to each participant. After 7 days of wearing the Actigraph, the accelerometer data was removed and analyzed using the ActiLife software (version 6.13.3). Non-wear time was defined as 20 min of consecutive zeros, which is commonly used to define non-wear in children [31]. The time spent in MVPA was determined using the Evenson cut point, which was derived using a 15-second epoch length [32]. Evenson cut-points [32] are applied to vertical axis data to calculate time spent in different PA intensities. Cut-off points (15 s -1) were < 25 counts for sedentary activity > 26 and < 573 for light physical activity (LPA), > 574 and < 1002 for moderate physical activity (MPA), and ≥ 1003 for vigorous physical activity (VPA). Time spent in MVPA was determined by adding minutes in a day of MPA and VPA. Cut-off point values are either divided or offer a fixed cut-point that can be used across age groups and have been found to have significantly better classification accuracy for MVPA [33]. The average time spent in MVPA per day and average daily wear time was computed using the data collected on each valid day. A valid day was defined as $\le$ 8 hours on weekdays and $\le$ 7 hours on weekends. For data to be included in the analysis, children were required to have worn the monitor for at least four days, including weekends [34].

### Fundamental movement skills proficiency

Data on FMS was collected using the Test of Gross Motor Development- Third Edition (TGMD-3) [35] to assess and describe the gross motor skills competence of the participants. The TGMD-3 is a standardized measuring tool to assess performance in gross motor skills qualitatively in the age range from 3 to 10 years of age. Four to five performance criteria of thirteen (13) different skills from the two subtests of motor skills are assessed in this test battery. The locomotor subset includes running, galloping, hopping, skipping, horizontal jumping, and sliding while the ball skills subset includes a two-hand strike of a stationary ball, one-handed forehand strike of a self-bounced ball, one-hand stationary dribble, two-hand catch, kick of a stationary ball, overhand throw and underhand throw. Mastery of the performance criterion in each skill is scored as 1 when the presence or mastery of that performance criterion is shown and a 0 indicates the absence of that performance criterion.

Two test trials were allowed for each test item and the scores were then added to provide a total raw score for each of the locomotor and ball skills items and subtests. The highest scores obtainable are 46, 54 and 158 points for the locomotor subtest, ﻿ball skills subtest and gross motor index (GMI) respectively. TGMD-3 standard scores, percentile ranks, age equivalents, confidence intervals, and GMI scores were determined. The subtest scaled score and the GMI were used for analysis to report the descriptive categories of each participant. Participants with subtest scaled scores of 1–3 and an index score of < 70 are classified as “impaired or delayed,” 4–5 and 70–79 as “borderline impaired or delayed,” 6–7 and 80–89 as “below average,” 8–12 and 90–109 as “average,” 13–14 and 110–119 as “above average,” 15–16 and 120–129 as “superior,” and 17–20 and > 129 as “gifted or very advanced/superior” [35].

The reliability coefficients for the total scale of the TGMD-3, i.e., locomotor and ball skills subscales have been reported based on three different sources of error variance: internal consistency which reflects the degree of similarity among the skills tested; reliability coefficients > 0.8 and low standard error measures suggests the TGMD-3 is a reliable test. Time sampling indicates a high magnitude of correlation (≥ 0.88) between the two trials which is an indication of its reliability in terms of stability over time. Reported inter and intra-score differences of 0.98 reveal a strong intertester reliability which suggests consistency of scores [35].

All the test items were assessed during one school day before and after the intervention period as well as 6 months later after no intervention took place. The intervention group were tested over two days because of the size of the group. The participants were grouped into three groups and rotated between 3 stations until all test items were assessed. Two trials of each skill were scored in person. Station one consists of locomotor skills (run, gallop, hop, skip, and slide). This station was set up in such a way that the tester could sit at a midpoint position while observing the execution of each skill from the side to score the execution. Station 2 consists of a wall station (one-hand forehand strike of a self-bounced ball, kick of a stationary ball, overhand throw, and underhand throw) and station three includes the two-hand strike of a stationary ball, two-hand catch, one-hand stationary dribble, and horizontal jumping. All TGMD-3 test items were assessed by trained senior researchers with a Kinderkinetics qualification. All stations had a demonstrator who demonstrated the activities for the participant; and a translator who provided understanding for those who were not clear on what was expected from them. Participants were also shown the correct execution of each skill on an IPAD.

### Intervention

The planning of the intervention and the intended outcomes of the intervention were guided by the use of the SPARK (Sports, Play & Active Recreation for Kids) Physical Education program written by McKenzie et al. [36]. The Curriculum and Assessment Policy Statement (CAPS) curriculum of SA was also taken into consideration in this planning [37]. The intervention involved nine weeks of program delivery, two times per week with each session lasting 30 min. The 18-session intervention was planned to improve the PA levels of young school children through improving motor competence (MC). The structure of each lesson included five minutes of warming-up exercises, 20 min of main activities, and five minutes of exercises to cool down. The sessions included activities to foster the skills that are assessed in the TGMD-3 [35]. As balance and aerobic fitness also hold a relationship with MC, such exercises were added, and aerobic fitness activities to increase the intensity level of PA participation. The programme was based on instructional learning with a focus on improving the technique of the skills and also improving PA. Each lesson was concluded with breathing exercises to relax the muscles before the participants returned to their classes. A detailed programme is published elsewhere [38]. To ensure maximum participation and cooperation, participants were grouped into smaller groups of five, and these groups were also changed weekly to also encourage socialization. Each activity station was timed with a blow of a whistle to ensure maximum delivery time at each session and to warrant that the planned lesson could be conducted to all participants in 30 min. The intervention was done outside on the school fields from the end of January to March which is a hot and rainy summer time. The researcher and four well-trained research assistants, all post-graduate students from the Department of Human Movement Science, delivered the FMS-based intervention as no PE programme was in place or PE teachers were available. A workshop was conducted by the researcher to train these research assistants before implementing the FMS-based programme and they were frequently updated on the planned lessons during the time of delivering the programme. Trained interpreters additionally translated the instructions from English to IsiXhosa to make sure that the participants understood the lesson content. Attendance was also recorded in an attendance logbook during each lesson to determine compliance with the program.

During the period of the intervention, the control group was engaged in their normal school routine or allowed to play freely during the PE timeslot as no PE teacher was appointed at the schools, and hence no programmes were conducted. The detailed programme that was followed and the apparatus that was used during the intervention was provided to the control schools after completing the intervention.

### Statistical analyses

All analyses were done using SPSS version 27 [39], which was used to analyze the results firstly through descriptive statistics (means, standard error, and standard deviations, or reported with 95% confidence intervals). Significant changes between the means across time were tested at the 0.05 alpha level. A hierarchical linear modeling analysis was followed to evaluate the interaction effects (sex, time, school, and group) of the intervention on the PA levels of the intervention group by comparing the group with the control group (*p* < 0.05). The Cohen’s-d cut-off points were used to interpret the practical significance of changes in the effect sizes for each analysis in this study: d ≥ 0.2 = small, d ≥ 0.5 = medium, and d ≥ 0.8 = large [40]. An estimated regression coefficient was also computed to evaluate the strength of the relationship between MVPA, and FMS competence. The strength of the relationship (r) was classified according to Cohen [40] (weak = 0.10–0.29; moderate = 0.30–0.49; strong = 0.50-1.0).

## Results

Seventy school children with the mean age of 7.12 years (± 0.71) (*n* = 35 IG; *n* = 35 CG), divided into an intervention (*n* = 35) and control group (*n* = 35), participated in the study. After the intervention group with a mean age of 6.96 (*±* 0.68) at baseline, underwent the motor intervention of 9 weeks 34 participants had complete Actigraph data (*n* = 17 boys; *n* = 17 girls) at retesting, representing a dropout rate of 2.9% in this group. Their PA was compared to the control group of thirty children (*n* = 14 boys; *n* = 16 girls, mean age 7.36 (*±* 0.68) with complete data sets at retesting (14.3% dropout from baseline) at baseline, directly after the intervention and six months later without receiving any intervention. The mean ages of the intervention and control groups at retesting, six months after the post-testing, were 7.61 (*±* 0.59) and 7.89 (*±* 0.75) years respectively. A high compliance of 97% with the intervention of 18 lessons, conducted over 9 weeks during PE periods, was recorded. The mean duration of PA monitoring in the study was 7.0 days, with an average wear time of 1337.14 min per day. Except for one participant wearing the Actigraph for 6 days, all the participants complied with the seven days of wearing the Actigraphs during all three time points. The descriptive data of the different intensities of PA and percentages of these PA intensities during the pre-test, post-test, and re-test according to group (intervention and control) are presented in Table 1. Descriptive outcomes (Means, Standard Error of the mean) of the locomotor skills and ball skills subsets and the gross motor index that were derived from these two skill domains as well as signifiance of changes over the three time points in these variables are additionally reported in Table 1.

No improvements were seen in the different intensities of PA per day or the percentage of time spent at these different intensities of the PA in the intervention group after completing the intervention (Table 1). The per-day SB, light-intensity physical activity (LPA), moderate-intensity physical activity (MPA), vigorous physical activity (VPA), moderate-to-vigorous-physical activity (MVPA), and percentages for all PA variables also showed very similar changes (increases or decreases) over time in both groups (Table 1). What was noteworthy, however, was that the LPA levels were visibly and significantly (< 0.05) higher than the SB, for both groups, with similar lowering trends in both groups overall three-time points.

**Table 1:** Descriptive outcomes of different PA intensities in the intervention and control group according to time

Variables	Intervention group						Control group					
	Pre-test (T1)		Post-test (T2)		Re-test (T3)		Pre-test (T1)		Post-test (T2)		Re-test (T3)	
	Mean	Std Dev./SE^#^	Mean	Std Dev. /SE^#^	Mean	Std Dev. /SE^#^	Mean	Std Dev. /SE^#^	Mean	Std Dev. /SE^#^	Mean	Std Dev. /SE^#^
PA levels												
SB(min/day)	144.3	14.65	195.7	17.85	147.9	11.10	144.8	15.71	197.8	18.92	148.0	12.05
LPA(min/day)	271.5	65.65	218.0	86.89	215.8	63.79	263.6	82.16	254.6	76.92	200.6	63.92
MPA(min/day)	42.7	15.89	33.4	15.67	32.5	12.48	44.8	16.95	42.9	16.03	38.6	16.35
VPA(min/day)	21.1	10.09	17.2	10.06	16.4	8.01	20.1	9.62	18.6	10.36	20.6	10.70
MVPA(min/day)	63.9	24.59	50.6	24.74	48.9	19.54	64.9	25.42	61.5	24.42	59.2	25.51
%SB	28.7	9.63	41.5	15.39	34.1	10.14	28.6	9.15	36.2	9.41	33.3	11.13
%LPA	48.8	7.02	40.1	10.34	45.5	6.92	48.0	7.85	43.1	6.37	42.7	7.06
%MPA	7.6	1.86	6.1	2.06	6.8	1.81	8.1	2.06	7.3	1.87	7.8	2.24
%VPA	3.7	1.48	3.1	1.50	3.4	1.44	3.6	1.35	3.1	1.40	4.2	1.59
%MVPA	11.3	3.08	9.2	3.39	10.2	3.02	11.7	3.17	10.4	2.96	12.0	3.53
LTRS	36.46	0.41#	43.74*	0.41#	44.56*	0.43#	33.66	0.52#	39.30*	0.53#	38.78	0.56#
BSTRS	41.79	0.56#	43.58*	0.56#	49.70*	0.59#	39.79	0.71#	42.60*	0.73#	48.63*	0.76#
GMI	103.91	0.89#	113.66*	0.89#	119.89*	0.93#	96.32	1.12#	106.87*	1.15#	111.03*	1.20#

SB = Sedentary behavior; LPA = Light−intensity physical activity; MPA = Moderate−intensity physical activity; VPA = Vigorous−intensity physical activity; MVPA = Moderate−to−vigorous−physical−activity; min = minutes; %= percentage; LTRS = Locomotor total raw score; BSTRS = Ball skills total raw score; GMI = Gross Motor Index; # = Standard Error; Std Dev = Standard Deviation, * significant improvement (*p*<0.05) from pre to post−test and post−test to rest

Table 2 displays the hierarchical linear modeling (HLM) results, showing the possible influences of different co-variants including school, sex (boys and girls), group (intervention and control), and time (pre-, post, and re-test). It also provides the means of the different PA variables of boys and girls separately in both groups over the three measuring points (Time (1), (Time (2), (Time (3). No group by sex by time effects were established that could reveal any positive effects of the program on any of the different PA levels of the IG (*p* > 0.05 for all PA intensities).

**Table 2:** Hierarchical linear modeling on the different PA intensities of the participants according to group, sex, and time

	Intervention group						Control group														
Varia-ble	Boys Mean			Girls Mean			Boys Mean			Girls Mean			Variance		p values						
	T1	T2	T3	T1	T2	T3	T1	T2	T3	T1	T2	T3	EST	School	Group	Sex	Time	Group*sex	Group* Time	Sex* Time	Group* Sex* Time
SB	146.1	196.5	147.7	142.4	194.9	148.1	144.6	195.3	146.2	145.0	200.3	149.7	0.000	0.01	0.73	0.80	0.00*	0.37	0.91	0.69	0.93
LPA	275.7	215.8	211.3	267.2	220.2	220.3	262.7	260.2	195.9	264.5	249.1	205.3	0.000	0.00	0.77	0.96	0.00*	0.96	0.01*	0.74	0.77
MPA	47.1	33.4	33.7	38.3	33.3	31.3	49.0	48.3	42.6	40.5	37.6	34.6	0.000	0.00	0.05*	0.04*	0.00*	0.38	0.15	0.60	0.37
VPA	23.9	17.0	17.4	18.4	17.3	15.4	23.2	21.8	24.9	17.1	15.3	16.4	0.000	0.00	0.41	0.01*	0.05*	0.22	0.09*	0.49	0.34
MVPA	71.1	50.5	51.1	56.7	50.6	46.6	72.2	70.1	67.5	57.6	52.8	50.9	0.000	0.00	0.11	0.02*	0.00*	0.28	0.18	0.60	0.35
Total PA	492.6	296.8	357.7	396.8	303.6	326.8	502.6	399.8	475.2	403.0	304.8	354.8	0.000	0.00	0.15	0.02*	0.00*	0.29	0.25	0.40	0.40

SB = Sedentary Behaviour; LPA = Light−intensity physical activity; MPA = Moderate−intensity physical activity; VPA = Vigorous−intensity physical activity; MVPA = Moderate−to−vigorous physical activity; TPA = Total s physical activity; EST = Estimate; T1 = Pre−test; T2 = Post−test; T3 = Re−test

Table 3 reports the statistical (p-values) and practical significance (d-values) or effect sizes of mean changes in PA intensities per day within the groups over the three time points. These results also revealed that changes in both groups, whether increases or decreases were again similar and ranged between small to large practical significant changes (*p* < 0.001; d = 0.1–0.9) in all PA levels over time for both groups (Table 3). A sex interaction effect (*p* = 0.017, Table 2) was, however, found in the MVPA levels of the groups. In the IG, a lowering trend was seen in the MVPA of the girls, with a slight but non-significant increasing trend in boys over time, specifically at T3, although the MVPA level was still lower compared to T1. However, the MVPA levels per day of the boys in the CG kept on decreasing over time (Table 3). Girls in the CG also showed a lowering trend similar to what was found in the IG of girls. Similar trends were also observed in the TPA of boys and girls in both groups. A sex interaction effect (*p* = 0.021, Table 2) shows MVPA decreases in boys and girls in both groups from T1 to T2 and increases from T2 to T3.

**Table 3:** Effect sizes of changes in pa levels per day (min/day) in both groups

	Intervention group					Control group			
Variables/Time	Mean diff (SD)	df		p-value	D	Mean diff (SD)	df	p-value	D
SB									
Pre-post (T1-T2)	52.25 ± 21.42	33	0.000*		3.4	51.53 ± 19.24	31	0.000*	3.5
Post-retest (T2-T3)	-48.98 ± 18.16	32	0.000*		3.2	-50.14 ± 20.62	26	0.000*	3.3
Pre-retest (T1-T3)	3.98 ± 18.77	33	0.226		0.2	4.51 ± 14.02	29	0.089*	0.2
LPA									
Pre-post (T1-T2)	-57.59 ± 54.72	33	0.000*		0.7	-2.86 ± 61.88	31	0.796	0.1
Post-retest (T2-T3)	-0.62 ± 84.00	32	0.967		0.0	-50.39 ± 80.84	26	0.003*	0.7
Pre-retest (T1-T3)	-53.25 ± 69.31	33	0.000*		0.7	-67.97 ± 77.37	29	0.000*	0.8
MPA									
Pre-post (T1-T2)	-10.25 ± 14.61	33	0.000*		0.6	-0.73 ± 12.73	31	0.749	0.1
Post-retest (T2-T3)	-0.33 ± 18.00	32	0.917		0.1	-3.32 ± 17.33	26	0.329	0.3
Pre-retest (T1-T3)	-10.09 ± 14.32	33	0.000*		0.7	-6.87 ± 15.25	29	0.020*	0.4
VPA									
Pre-post (T1-T2)	-4.44 ± 8.97	33	0.007*		0.4	-1.28 ± 7.15	31	0.319	0.2
Post-retest (T2-T3)	-0.59 ± 11.35	32	0.766		0.1	1.82 ± 12.27	26	0.447	0.2
Pre-retest (T1-T3)	-4.74 ± 8.27	33	0.002*		0.5	0.08 ± 9.90	29	0.966	0.1
MVPA									
Pre-post (T1-T2)	-14.69 ± 22.94	33	0.000*		0.6	-2.01 ± 19.13	31	0.557	0.2
Post-retest (T2-T3)	0.92 ± 28.91	32	0.856		0.1	-1.50 ± 28.38	26	0.786	0.1
Pre-retest (T1-T3)	-14.83 ± 21.68	33	0.000*		0.6	-6.80 ± 23.99	29	0.132	0.2

Mean diff = Mean difference; df = Degree of freedom, d = Cohen d; SB = Sedentary behaviour; LPA = Light physical activity, MPA = Moderate physical activity; VPA = Vigorous physical activity; MVPA = Moderate−to−vigorous physical activity; d > 0.2 = small, d > 0.5 = medium and d > 0.8 = large; (− Sign) = a reduction in mean value per day; * = significant; T = Test; T1 = Pre−test; T2 = Post−test; T3 = Re−test

Table 4 reveals the total number of boys and girls in the groups who met the WHO recommendation for daily MVPA while Table 5 reports these results of daily MVPA according to group, time, and sex based on WHO recommendations.

**Table 4:** Classification of MVPA stratified by group and time based on the WHO recommendation

	Intervention group control group					
MVPA classification	Combined (boys + girls; *n* = 35) N (%)			Combined (boys + girls; *n* = 35) N (%)		
	T1	T2	T3	T1	T2	T3
Combined > 60 min/day	21 (60)	12 (35.3)	9 (26.5)	19 (54.3)	16 (50)	14 (46.7)
Combined < 60 min/day	14 (40)	22 (64.7)	25 (73.5)	16 (45.7)	16 (50)	16 (53.3)

N = number of participants; % = Percentage

Overall, a sizable and similar number of children in the IG and CG groups met the recommended daily 60 min at pre-test (T1) (60% in the IG and 54.3% in the CG, Table 4), although sex differences were observed. These were 82.4% of boys and 38.9% of girls in the IG group, compared to 64.7% and 44.4% of boys and girls of the CG group (Table 5). At the end of the post-test and re-test, the percentages of participants who met these WHO recommendations were, however, higher in the CG (50%, 46.7%) than in the IG (35.3%, 26.5%) (Table 5). A big decrease in the number of boys in the IG compared to in the CG that met the daily MVPA guidelines at post-test and re-test (IG 37.5%, 35.3% vs. CG 58.8%, 64.3%) was found. Similar trends were observed for girls in the IG and CG groups (IG 33.3%, 17.6% vs. CG 40%, 31.3%) (Table 5).

An additional estimated regression coefficient analysis was done to determine possible association between MVPA levels and the different aspects of fundamental motor skills as displayed in Table 6, which could have been attributed to the intervention effects. Table 6 displays these correlational coefficients between MVPA in the IG, CG, and motor skills for data of the three time points period (pre-, post-, retest). These correlations were calculated with SAS PROc SURVEYREG where the dependency of measurements on the same person was taken into account. A complete discussion of the effect of the intervention can also be found elsewhere (Idamokoro, 2023). Although both the locomotor skills (LSTRS), ball skills (BSTRS) Gross motor Index (GMI) improved significantly as a result of the intervention (Table 1), only one significant association could be established between MVPA and these FMS variables (Table 6). This association was, however, weak but negative between the BSTRS (*R* = -0.22; *p* = 0.043) and MVPA and was also only evident in the girls of the IG group (Table 6).

**Table 5:** Classification of MVPA according to group, time, and sex based on the WHO recommendation

	Intervention group						Control group					
MVPA classification	Boys (*n* = 17) N (%)			Girls (*n* = 18) N (%)			Boys (*n* = 17) N (%)			Girls (*n* = 18) N (%)		
	T1	T2	T3	T1	T2	T3	T1	T2	T3	T1	T2	T3
> 60 min/day	14 (82.4)	6 (37.5)	6 (35.3)	7 (38.9)	6 (33.3)	3 (17.6)	11 (64.7)	10 (58.8)	9 (64.3)	8 (44.4)	6 (40)	5 (31.3)
< 60 min/day	3 (17.6)	10 (62.5)	11 (64.7)	11 (61.1)	12 (66.7)	14 (82.4)	6 (35.3)	7 (41.2)	5 (35.7)	10 (55.6)	9 (60)	11 (68.7)

N = number of participants; % = Percentage

**Table 6:** Relationship between MVPA and motor skills according to group and sex

Variable	Intervention group (*n* = 57)		Boys (*n* = 27)		Girls (*n* = 30)		Control group (*n* = 36)		Boys (*n* = 17)		Girls (*n* = 19)	
	R	p-value	R	p-value	R	p-value	R	p-value	R	p-value	R	p-value
LTRS	-0.26	0.05	-0.35	0.094	-0.13	0.452	-0.17	0.08	-0.04	0.765	-0.04	0.605
BSTRS	-0.04	0.677	0.06	0.682	-0.22*	0.043	0.09	0.411	0.09	0.589	0.10	0.536
GMI	-0.11	0.296	-0.11	0.431	-0.07	0.621	-0.15	0.157	-0.05	0.771	-0.05	0.715

LTRS: Locomotor Total Raw Score, BSTRS: Ball Skill Total Raw Score, GMI: Gross Motor Index, MVPA: Moderate−to−vigorous physical activity; Correlation values (r) of 0.10–0.29 (weak), 0.30–0.49 (moderate) and 0.50–1.0 (strong); *p < 0.05

## Discussion

The main objective of this study was to appraise the immediate and sustainable effects of motor intervention on the different PA intensities, more specifically the MVPA level of 7 to 8-year-old Eastern Cape school children growing up in disadvantaged areas while also determining possible associations between their MVPA levels and FMS. This was done against the supposition that improved motor skills as a result of the intervention might contribute to higher PA levels. The main findings, however, reveal no intervention effects that show either an immediate or a sustainable effect on improving PA behavior among these children with limited resources to apply their motor skills in their everyday lives. MVPA was also not positively associated with the FMS in the intervention group, which could confirm a relationship between MVPA and MC. Overall, these finding agrees with the conclusions in the review paper of Barnett and colleagues [41], stating that the degree of the relationship between PA and gross motor competence is still not clear and needs more understanding. Inconclusive relationships between PA and MC, are also reported in a most recent review paper [18] which concurs with our findings. A better understanding of the activity behavior and interests of children living in disadvantaged and rural areas is therefore especially needed to improve their PA behavior, at least through the content of movement programmes, as suggested by researchers [42].

Although the PA behavior of both groups was very similar at the pre-test, changes over time revealed higher (*p* > 0.05) MVPA in the control group at the post-test and re-test. At the post-test, just after completion of the intervention that required high levels of activity from the intervention group, increases were found in their SB while also reducing all other activity intensities (light, moderate, vigorous, and MVPA). One plausible explanation could be that the intervention group was reacting to the “effects of compensatory” changes during the post-test just after completing the intervention. Such compensatory changes are described as when children tend to compensate for increased PA levels on one day by decreasing their activity levels the following day [43]. As the children in the intervention group had never been exposed to an intervention of this nature that required high physical activity levels, overdose effects might have occurred, as also reported by other researchers [44]. A slight recovery effect was, however, observed in the MVPA of the boys in the intervention group during the retest, which was encouraging. The MVPA of the control group, however, also lowered, but to a smaller extent, at the post-test and re-test compared to what was observed in the intervention group. Further analysis of the MVPA levels on an individual level of each participant revealed high daily MVPA levels in three of the boys in the control group at post-test and re-test (MVPA = 96.24 min, 95.95 min, 93.79 min, and 108.4 min, 97.1 min, and 93.64 min) compared to the mean value of the control group [61.5 min (post) and 59.2 min (retest)], which could have contributed to the higher MVPA levels of this group. Although informal discussions after completing the study revealed that all these boys were in a local team where games like cricket and soccer were played daily, they were also involved in street soccer. In the intervention group that was located in a more urban environment, none of the participants were involved in similar after-school activities or high-intensity activities. The similar SB patterns that were found in both the groups at post-test testing can, however, not be fully explained by compensatory changes but could have been influenced by the school schedule such as the assessment periods at the end of March which could have required the children to sit more and study when they were wearing the Actigraphs. This is also a rainy season in the area and such weather but also very hot conditions could also have restricted outdoor and more vigorous play at home in both groups. The dwellings of these children are also often in densely populated areas with small houses and yards which might also restrict movement of a more vigorous nature when at home. Although suggestive, it can also be that active transport over long distances to school in the remote and mountainous setting where the control group school is located might have also contributed to the stable MVPA levels that were found over time in this group. In agreement, it is reported that the MVPA of children in these communities is rather accumulated from habitual activities such as house chores [45] and possibly walking to school [46]. These findings also concur with the zero associations and even a negative association that were found between ball skills and MVPA in the girls of the intervention group. It is, however, consistent with the findings on preschool children in Australia [47], which also only showed a negative association between MVPA and ball skills in girls. A positive association was, however, reported between ball skills and MVPA of boys, which was different from our findings.

The percentage of boys and girls in the control group who met the daily MVPA recommendation did not change profoundly compared to the much fewer boys and girls in the intervention group who met this recommendation over time. More boys, however, met the recommended daily guidelines for MVPA than girls in both groups, which is consistent with the findings of other researchers on similar-aged children worldwide [48] but also in SA [24, 49, 50]. A South African study [50] on 6–8-year-old children revealed 80.0 min of daily MVPA, while another study [24] on children in grades 1–4 revealed 82.1 daily minutes of MVPA compared to the lower 63.9 min and 64.9 daily MVPA minutes in our groups during the pretest (Table 1). In agreement with our findings, a study involving 650 primary school children (10–15 years) in Port Elizabeth found that 40.8% did not reach the recommended MVPA minutes per day [15], which is similar to the 40% and 45.7% of participants in both groups in the current study that also did not comply with this recommendation during pre-testing. The time spent in LPA by both groups was, however, much higher compared to the time spent in SB per day. This is different from studies that usually report higher SB levels [24], although similar to the findings of same-aged children with similar SES backgrounds in SA [50]. It is also consistent with other studies in Sub-Saharan Africa, where children spend more time in LPA than in MVPA [51–53]. This finding, however, reveals that these children are less inactive (at least on a sedentary level) compared to other similar-aged children in other countries or non-rural and higher SES settings. This again suggests unique PA behavior in different settings that needs further attention from researchers.

Higher percentages of participants also met the recommended MVPA guideline at the pre-test, with clear decreases at the post-test and the re-test. One plausible explanation could be that at the beginning of the school year, during the pre-test, the energy levels and motivation of the children were high, which might have been conducive to their higher activity levels. As the school term progressed, at post-test and re-test, fatigue might have set in as they were in a period of termly assessments, while weather-related changes such as very hot or rainy conditions might have restricted them from outside play during the period of data collection. In addition the hot day temperatures might also have restricted their effort, especially partaking in aerobic activities as the intervention was done outside on the playing field. Break and play periods were also restricted. In support of these findings, it is generally reported that poor school infrastructure, large class sizes, a lack of motivation and commitment, and weather and health conditions are factors that may affect the PA levels of school children [46, 54].

Although the intervention group showed a slight recovering effect during the retest in their activity levels as their sedentary behavior decreased slightly with a slightly higher time spent in LPA and MVPA, the control group still spent more time in MVPA from pre-test to re-test compared to the intervention group. The school of intervention is located in a more urban setting which might require less activity such as commuting long distances to school compared to the CG. The estimated regression coefficients, however, also confirmed that MVPA and the motor skills of the group were not associated, which agrees with the inconclusive relationships between PA and MC, specifically object control and locomotor skills that are also reported in a recent review paper [18]. This finding was still surprising because it was assumed that the motor programme followed by the intervention group could facilitate their PA levels based on the premise that the improved motor competence found as a result of the intervention might have contributed indirectly to higher PA levels, which was not the case. It, therefore, became clear that if changes in PA behavior are strived towards as part of a motor skills programme, the intervention needs clear individualised activity goals, such as including educational knowledge about the importance of regular PA and how to apply and transfer the content of motor programs to their daily lives, which was not a direct objective of our intervention. Findings like these are, however, not uncommon, even while focusing on changing these behaviors. A review study including 129 studies that examined the effectiveness of school-based interventions based on changes in psychological determinants, PA, and health outcomes in 6-19-year-old children, revealed that 6.8% of the studies also experienced a reduction in PA in the intervention groups compared to the control groups after participating in the intervention. The researchers [14], believe that these negative effects might have resulted from increased psychological pressure from the intervention, which again might have contributed to an aversion towards PA after the intervention.

Consistent with our findings, a 2-year trial study on grade 3 to 5 children in rural Nebraska-Kearney, aiming to attenuate obesity and improve PA, also revealed no differences between intervention and control groups. This intervention, which aimed to increase energy expenditure in the PE classroom to influence increases in PA outside of the classroom, instead showed significantly more PA outside of school in the control group compared to the intervention group (a 20% increase versus a 16% lowering effect in the intervention group). A plausible explanation was that the mandatory increase in classroom PA of the intervention group acted as an overdose that caused decreases in PA outside of the PE classroom [44], which is aligned with the explanation of compensatory reactions after high-intensity activities [43]. A study of school children aged 8–11 from Hong Kong Island also revealed similar findings [55] where the percentage MPA and VPA decreased between the pre-test and the 6-month follow-up periods in all the groups. The control group also spent higher percentages in MPA (12.1 ± 10.8 and 10.7 ± 9.3) and VPA (2.9 ± 3.1 and 2.1 ± 3.8) from pre-test to re-test compared to the intervention group (8.9 ± 8.0 and 7.2 ± 7.0), (2.5 ± 2.0 and 1.9 ± 2.4). The various prompts that were given to change the participants’ health behaviors were found to be largely ineffective, possibly because the information received from the educational program had not been personalized to the participant’s knowledge level, cognitive abilities, interests, or behavior patterns. Another plausible explanation provided for the decreases found in both moderate and vigorous physical activity from pre-test to re-test in our groups was that it might be related to age- and maturity-related declines, which have been observed in PA [55]. Decrease and non-significant sustained results for both groups (intervention and control) at follow-up were also reported in another study [56]. One of several explanations that were offered was that intervention periods shorter than 12 months might be insufficient.

Interventions in primary schools that have reported sustained intervention effects were mostly over longer periods of 3 to 6 school years with more intensive intervention programs [56]. Additionally, factors such as variations in PA due to different seasons and shifts in development implying a decrease in PA as an individual’s age suggest that children do not possess a fixed threshold for PA [56]. In several interventions that have reported significant intervention effects for PA, the differences between the intervention and control groups also narrowed in magnitude over time [57, 58]. Therefore, further research is suggested to investigate the best approach to creating sustained long-term changes in PA as children progress through childhood, adolescence, and adulthood, and longer periods of follow-up testing are recommended.

According to Demetriou and Honer [14], the nature, content (a framework that addresses specific needs), and duration of interventions also need consideration to improve outcomes. It might be that the 9-week duration, including 18 lessons in the current study, was too short to show improvements. Additionally, the lack of a clear focus on changing PA behaviors through the content of the program to obtain desired changes in the MVPA levels might also have influenced the findings of this study. The MVPA levels of the IG could not be sustained after the intervention, during PE classes at school, or in after-school activities because of a lack of continued exposure to similar content that they were exposed to during the intervention. After completion of the intervention, the PE classes were again used as before, for activities such as food breaks, refreshments, or a time to catch up on academic activities by slower learners. The classroom teachers were also not PE specialists and did not consider themselves able or motivated to teach PE-specific content classes. The impact of PE specialists and a high-quality program on children’s PA experiences should therefore be highlighted to influence policymakers and teacher education providers. A clear lack of consideration for the importance of PE and consequently investing in time for children to engage in PE programs was therefore evident in the school environment of the intervention school. Moreso, these children also lived near the school; therefore, they did not have to walk far to school or were even transported to school, which might have contributed to their higher inactivity levels. Additionally, children in the IG were also not exposed to local sports teams. Seasonal differences in PA (very hot or rainy periods) may also have contributed to the decreases in PA levels of both groups as also reported by other researchers [56].

Overall, these findings confirmed that the degree of the relationship between PA and gross motor competence still needs more understanding from researchers. Our findings also confirmed unique PA patterns in children raised in disadvantaged and more rural settings which require a better understanding of the activity behavior and interests of such children to improve their PA behavior through the content of movement programs. In this regard, it is reported [41] that different types of PA may have differing associations with skills and that it is feasible that participation in the types of activities that use particular skills may lead to higher associations with that type of skill competence.

Regular participation in PE programs could help to create a wide range of available PA opportunities that would help children increase the duration and frequency of daily MVPA within the school setting. It might also be a specific strategy in disadvantaged and rural school settings to strive towards shifting or re-allocating light activity to MVPA levels. García-Hermoso and colleagues [59], reported in this regard that when SB can be shifted towards MVPA, adiposity can be addressed positively.

### Study strengths

The strength of this study is that it was the first study to use accelerometry to objectively record the levels of PA in primary schoolchildren in rural schools of the Eastern Cape for 7 days over three time points. This created an opportunity to not only study the PA levels of these children but also to determine possible carry-over effects of the motor intervention on PA on a sustainable level. Furthermore, this study is also the first to be conducted in the rural context of SA, seeking to assess the possibilities of a motor program to improve the levels of PA in children. The insights gained from this study provided valuable understanding regarding the challenges that schools in these settings encounter while incorporating PE and, subsequently, motor programs into their daily academic schedule. The environmental challenges related to fostering children’s adherence to the prescribed WHO PA guidelines for children were also noted.

### Study limitations

This study also had limitations that also need to be acknowledged. Due to natural fluctuations, a 7-day assessment period may not fully represent the typical PA levels of children and may still be too short and dependent on weather and health-related conditions during the assessment period. More insight into the activities that these children engage in during the seven days of Actigraph wear could also have been obtained if the parents had completed an activity diary for the seven days when they wore the Actigraphs. Differences in measurement tools when compared to other studies could also limit the findings. If psychological factors influencing PA, such as knowledge, self-concept, attitudes, and motivation, could have been investigated, it could have provided further insights into comprehending why the participants’ PA levels declined over time. Future studies should investigate the extent to which these influences can mediate the effects of an intervention on the PA levels of school children. Additionally, the influence and challenges faced by parents regarding PA outside the school environment were not examined, and future research should consider these influences. The intervention might have been too short, although it was designed according to the school terms of South African schools. It is, however, recommended that studies with similar objectives should include a bigger number of participants and be longer in duration, at least 12 months and above, to obtain more desirable results.﻿

## Conclusions

This intervention strived to improve the PA levels of young children through an intervention that focused on addressing the motor competence of young children and thereby indirectly motivating them to use this improved motor skills repertoire to live more active lives. This strategy, however, proved not to be successful in improving or sustaining the PA levels of the participants. It was clear from the findings that much more understanding is needed of the PA behaviours of these children, to be able to address it accordingly. Therefore, more insights are needed and required on how to address and specifically sustain positive PA changes in young children growing up in poor SES environments. These insights have the potential to address their specific challenges in living active lives. Strategies should also be developed and implemented to support schools in investing time in PE classes and also inside the classroom, which will improve PA outside the classroom. Unfortunately, the reality is that interventions of this nature where researchers deliver the programs will not be sustainable without the necessary buy-in of schools, including the school board, the headmaster, and the teachers. It is therefore worthwhile to invest and test other action plans and conduct further research in schools that are situated in rural and socioeconomically disadvantaged areas that may offer solutions to the questions raised by the current study.

## Data Availability

The dataset is the property of the North-West University under the supervision of Anita E. Pienaar. In this regard, Anita E. Pienaar should be contacted if, for any reason, the data included in this paper needs to be shared. Anita E. Pienaar is the principal investigator of this study.

## Abbreviations

BSTRS: Ball Skill Total Raw Score
CG: Control group
GMI: Gross Motor Index
IG: Intervention group
LPA: Light physical activity
LTRS: Locomotor Total Raw Score
MPA: Moderate physical activity
MVPA: Moderate-to-vigorous-physical activity
VPA: Vigorous physical activity
PA: Physical activity
TGMD-3: Test of Gross Motor Development-3
